# A Prospective Study of the Assessment of the Efficacy of a Biodegradable Poly(l-lactic acid/ε-caprolactone) Membrane for Guided Bone Regeneration

**DOI:** 10.3390/jcm12185994

**Published:** 2023-09-15

**Authors:** Rena Shido, Seigo Ohba, Risa Tominaga, Yoshinori Sumita, Izumi Asahina

**Affiliations:** 1 Department of Regenerative Oral Surgery, Nagasaki University Graduate School of Biomedical Sciences, 1-7-1 Sakamoto, Nagasaki 852-8588, Japan; r-shido@nagasaki-u.ac.jp (R.S.); risaompm@tmd.ac.jp (R.T.); izumi.asahina@icloud.com (I.A.); 2 Center for Oral Implant, Nagasaki University Hospital, 1-7-1 Sakamoto, Nagasaki 852-8588, Japan; 3 Department of Psychosomatic Dentistry, Graduate School of Medical and Dental Science, Tokyo Medical and Dental University, 1-5-45 Yushima, Tokyo 113-8510, Japan; 4 Department of Medical Research and Development for Oral Disease, Nagasaki University Graduate School of Biomedical Sciences, 1-7-1 Sakamoto, Nagasaki 852-8588, Japan; y-sumita@nagasaki-u.ac.jp; 5 Department of Oral and Maxillofacial Surgery, School of Medicine, Juntendo University, 3-1-3 Hongo, Tokyo 113-8421, Japan

**Keywords:** guided bone regeneration (GBR), poly(l-lactic acid/ε-caprolactone) membrane, biodegradable material, alveolar bone augmentation, collagen-based membrane, carbonate apatite

## Abstract

Biodegradable guided bone regeneration (GBR) membranes consist primarily of collagen and aliphatic polyesters. This study assessed the comparative efficacy of a poly(l-lactic-caprolactone) [P(LA/CL)] membrane versus that of a collagen membrane in GBR. Patients requiring GBR simultaneously or before dental implant placement in edentulous regions were randomly assigned to one of two membranes. Within each membrane, they were subdivided into 3 groups: dental implants were placed simultaneously with GBR in groups A and B, and 180 days post-GBR in group C. The augmented bone width was measured at 1, 3, and 6 mm from the implant’s neck (groups A and B) or the reference line (group C), utilizing cone-beam computed tomography images, immediately and 150 days post-surgery. A histological study was performed to evaluate bone formation in group C. No adverse events were observed. In the collagen group, the absorbed ratios of the augmented bone were 40.9 ± 36.7%, 29.4 ± 30.1%, and 11.1 ± 22.0% at 1, 3, and 6 mm, respectively; the ratio at 6 mm was significantly lower than that at 1 mm (*p* = 0.0442). In the P(LA/CL) group, those were 26.2 ± 27.3%, 17.1 ± 19.7%, and 13.3 ± 16.4% at 1, 3, and 6 mm, respectively, with no significant difference at each point. No significant inter-membrane differences were observed. The bone augmentation potential of the P(LA/CL) membrane matched that of the collagen membrane. P(LA/CL) could be used as a safe and effective membrane in GBR.

## 1. Introduction

Guided bone regeneration (GBR) is an essential technique to achieve effective bone augmentation for the placement of dental implants in the optimal position in contemporary dental implant treatment. It requires prosthetic-driven placement of dental implants in cases where the alveolar bone volume is insufficient [1]. The augmented region is enveloped by a barrier membrane (GBR membrane) that precludes the infiltration of gingival mucosa-derived cells that interfere with osteoblastic bone regeneration [2]. There are several crucial properties of the GBR membrane, such as biocompatibility, space-making, occlusivity, and barrier to prevent fibroblastic cell migration [3,4,5].

There are two types of GBR membranes: biodegradable and non-biodegradable [5,6]. Some studies have compared the bone augmentation capability of GBR between these two types of membranes. Titanium mesh and polytetrafluoroethylene with titanium membrane TF are mainly used for the non-biodegradable (non-absorbable) membranes, which are characterized by the ability to maintain their initial physical properties. Whereas collagen-derived membranes are commonly used for biodegradable (absorbable) membranes. Some studies reported that non-biodegradable membranes showed superior capability in terms of bone augmentation [7], whereas others found no significant difference between them [8]. In contrast, non-biodegradable membranes require additional surgical intervention for removal after bone healing, while biodegradable membranes do not, which is advantageous for both patients and clinicians [9].

Biodegradable GBR membranes are mainly composed of collagen or aliphatic polyesters [10,11]. Although animal-derived materials, such as collagen, are currently considered safe, unknown pathogens may contaminate them. Furthermore, biodegradable membranes might not be absorbed in an appropriate period to achieve bone augmentation after GBR [3] because it is difficult to control the resorption rate of natural products such as collagen. Therefore, bone augmentation may not be achieved as planned. Consequently, in recent years, biodegradable synthetic polymeric materials have been used as GBR membranes owing to their controllable absorption rates in vivo [12]. However, the sample size was inadequate to provide sufficient data.

The copolymer of L-lactide and caprolactone [P(LA/CL)], a biodegradable synthetic polymer material that characteristically has elasticity, has been reported to serve as an effective barrier against cell invasion and is absorbed in vivo at an appropriate time for GBR [13]. In addition, the P(LA/CL) membranes have the unique feature of extensibility. Our pilot study using a GBR membrane composed of P(LA/CL) (Cytrans^®^ elashield, GC Corp, Tokyo, Japan) demonstrated its safety for GBR [14]; however, the limited sample size precluded its efficacy. Therefore, this study aimed to assess the safety and efficacy of the P(LA/CL) membrane for GBR by comparing it with the collagen membrane. Moreover, in the previous study [14], the included cases were limited to cases in which the P(LA/CL) membrane was only used for GBR concurrent with implant placement. In this study, however, cases that required alveolar bone augmentation by GBR before implant placement were also included to evaluate larger bone defects.

## 2. Materials and Methods

***Patients:*** Patients who needed to undergo GBR simultaneously or before the placement of a dental implant in the edentulous regions between January 2021 and March 2022 at Nagasaki University Hospital were included in this study. The P(LA/CL) membrane used in this study was previously assessed for safety in the first clinical trial on five patients as a pilot study [14]. In this study, the sample size was set at 20 cases because it was conducted as an exploratory study of the efficacy and safety of the P(LA/CL) membrane following the previous pilot study. All candidates were matched using the following criteria:

Inclusion criteria:

(1) Patients requiring GBR because of insufficient bone volume in the edentulous region where dental implants are required to be placed.
(2) Age range: 20–90 years.
(3) Patients who understood the consent document and agreed to participate in the study at their discretion.

Exclusion criteria:

(1) Patients suffering from severe hematological disorders.
(2) Patients with abnormalities in the organs of calcium metabolism, such as the kidneys and digestive organs, or suspected connective tissue diseases.
(3) Patients who were difficult to follow up during the study for various reasons.
(4) Patients whose social and domestic circumstances precluded compliance with the requirements of this study.
(5) Smokers.
(6) Patients in need of surrogates.
(7) Patients deemed to be inappropriate for participation in this study.

**Figure 1:**
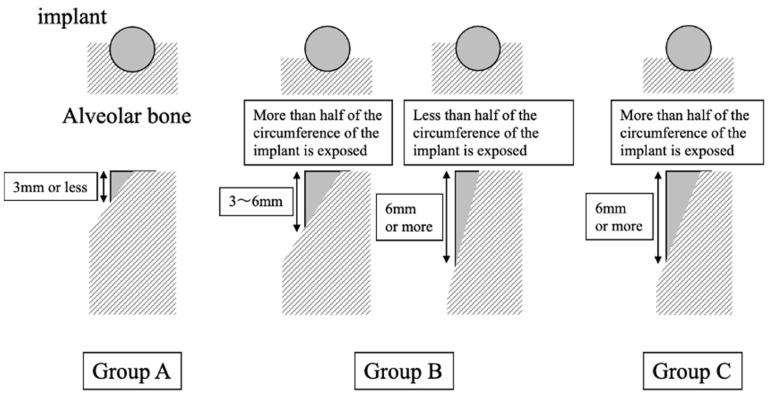
The groups according to the implant exposure.

***Regions:*** Implant placement simulations using software (SimPlant Pro^®^ 18.0, Dentsply Sirona Implants, Charlotte, NC, USA) were performed using cone-beam computed tomography (CBCT) data acquired within 6 months prior to enrollment. Patients who required bone augmentation by GBR for placement of implants in the ideal prosthetic position and direction were included in this study. Patients were divided into three groups according to the implant exposure, which was predicted by the simulation (Figure 1), to prevent the selection of the collagen and P(LA/CL) groups.

*Group A:* The implant exposure was less than 3 mm vertically, and less than half of the implant was horizontally.
*Group B:* (1) The implant exposure was between 3 and 6 mm vertically and horizontally, with more than half of the implants. (2) Implant exposure greater than 6 mm and less than half of the implant horizontally.
*Group C:* Implant exposure was more than 6 mm vertically and horizontally in more than half of the implants, in which case primary stability was not considered to be obtained. In this group, the first step was bone augmentation by GBR, and implant placement was performed during the second surgery.

The GBR membrane was randomly assigned as either a collagen-based membrane (Bio-Gide^®^, Geistlich Pharma, Wolhusen, Switzerland) (collagen group) or a P(LA/CL)-based membrane (Cytrans^®^ elashield) [P(LA/CL) group]. 

### 2.1. Surgical Procedures (Figure 2)

All surgical procedures were performed under local anesthesia by two oral and maxillofacial surgery specialists. The bone graft material consisted of a 1:1 mixture of carbonate apatite (Cytrans^®^ Granule M, GC Corp) and autogenous bone in volume ratio. The autogenous bone was harvested by the bone scraper (safe scraper^®^, META, Reggio Emilia, Italy).

*Groups A and B:* The mucoperiosteal flap was elevated and alveolar bone atrophy or defects were observed. Following implant placement using a surgical guide, the exposed implant region was covered with a bone graft material mixture, and the materials were enveloped by the GBR membrane. The GBR membrane was fixed with tuck pins (tru TACK^®^, ACE surgical, AZ, USA) to maintain the graft materials at the place in group B but not in group A. After a tension-reducing periosteal incision was made, the wound was closed without tension.
*Group C:* Bone augmentation was performed as in group B but without implant placement.

- Bone augmentation using GBR was performed simultaneously with implant insertion in groups A and B.
- Bone augmentation by GBR was performed prior to implant insertion in group C.
- CT images of group C (before, left, right, middle, and 150 days after GBR; right).

**Figure 2:**
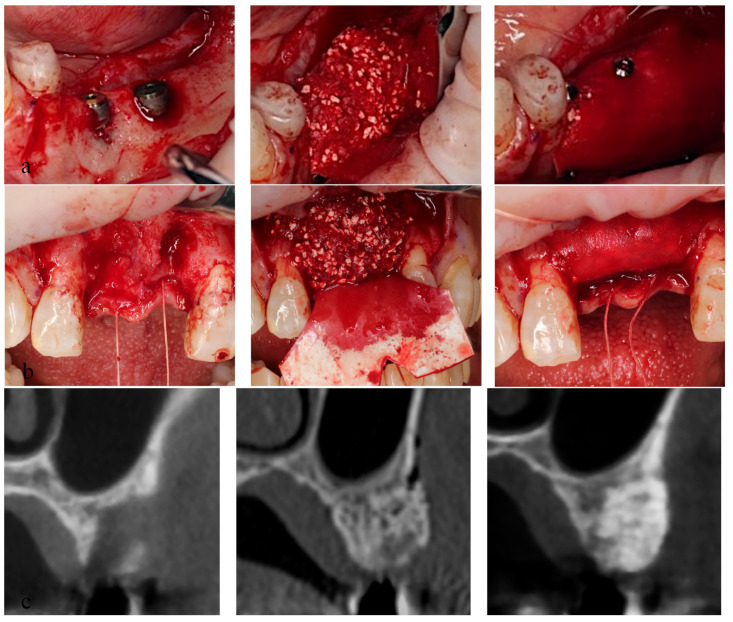
Surgical procedures.

### 2.2. Follow-Up

Patients were continuously followed up at 30 ± 7, 60 ± 7, and 90 ± 7 days after GBR. Secondary surgery was performed 150 ± 14 days after dental implantation with GBR in groups A and B. Simultaneously, the implant stability quotient (ISQ) was evaluated (Osstell ISQ^®^, Osstell AB, Götheborg, Switzerland) and CBCT was performed immediately after the second surgery. In group C, the CBCT examination was performed 150 ± 14 days after GBR, and implants were placed with simultaneous bone biopsy 180 ± 14 days after GBR. 

### 2.3. Assessment

**Figure 3:**
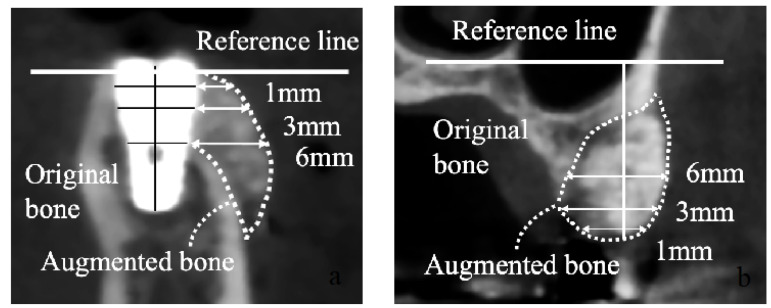
CT evaluation. The implant platform was selected as the reference line in groups A and B (**a**). The nasal floor and mandibular margin were selected as reference lines for the maxilla and mandible, respectively, in group C (**b**).

(1) Adverse events: Events that could be related to the GBR membrane were identified as adverse events that occurred during the study period from GBR to the second surgery.
(2) CT evaluation: The augmented bone volume was assessed based on CBCT images, according to a previous report [14] (Figure 3), at two points: immediately after (T1) and
(3) 150 ± 14 days after GBR (T2). The absorbance rate of the augmented bone was calculated using T1-T2/T1.
  *Groups A and B:* The platform level was designated as the reference line. The augmented bone width was calculated horizontally at 1, 3, and 6 mm from the reference line.
  *Group C:* The nasal floor and mandibular margin were designated as the reference lines for the maxilla and mandible, respectively. The augmented bone height was calculated from the reference line to the edge of the augmented bone. The alveolar bone width was calculated at 1, 3, and 6 mm from the reference lines.
(4) ISQ values: The ISQ values were evaluated during the second surgery using Osstell in groups A and B. The evaluation was performed on both the labial/buccal and lingual/palatal sides. The average ISQ value of both sides was determined as the ISQ value of the implant.
(5) Histology of the bone biopsy specimen in group C: To evaluate the bone tissue area, Villanueva–Goldner staining was performed (Kureha Special Laboratory, Fukushima, Japan). The bone tissue area was analyzed using the ImageJ software (NIH, Bethesda, MD, USA). The ratio of the bone tissue area to the total specimen obtained by biopsy was measured by combining the green area indicating mature bone and the dark red area indicating osteoids as the bone tissue area.

### 2.4. Statistical Analysis

Each value was compared between groups and time points, and a *p*-value < 0.05 was considered statistically significant. It was conducted using the paired *t*-test was performed for statistical assessment to compare T1 and T2 in each group, and the student *t*-test to compare two groups, which makes no specific assumptions about the distribution of the population.

## 3. Results

### 3.1. Patients and Sites

A total of 25 sites from 20 patients were included in this study. Participants’ ages ranged from 41 to 78 years (mean: 60.8 years). There were seven, ten, and eight sites in groups A, B, and C, respectively. The details of the membranes are listed in Table 1.

### 3.2. Adverse Events

No adverse events related to GBR or implants were observed in any patient. None of the patients in group C required additional bone augmentation at the time of implant placement.

### 3.3. ISQ Values

The ISQ values were 77.8 ± 3.3 and 79.2 ± 1.6 in the collagen and P(LA/CL) groups, respectively, in group A (*p* = 0.5519). The ISQ values were 74.9 ± 8.4 and 78.1 ± 4.0 in the collagen and P(LA/CL) groups, respectively, in group B (*p* = 0.5229). In groups A and B, the ISQ values were 76.2 ± 6.4 and 78.4 ± 1.6 in the collagen and P(LA/CL) groups, respectively (*p* = 0.4252). In group C, the ISQ values were 80.0 ± 7.8 and 75.8 ± 3.0 in the collagen and P(LA/CL) groups, respectively (*p* = 0.5776). No significant differences were observed between the groups.

### 3.4. CT Evaluation

#### 3.4.1. Group A 

**Figure 4:**
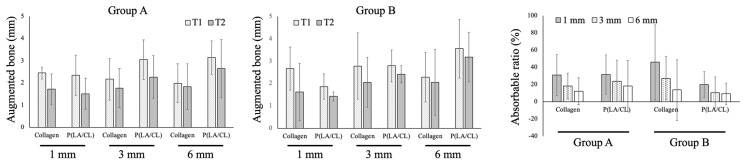
CBCT examination in groups A and B. The augmented bone widths did not change significantly from T1 to T2 in either group.

T1-T2 of the augmented bone width at 1 mm from the reference line was 0.73 ± 0.50 and 0.83 ± 0.83 mm in the collagen and P(LA/CL) groups, respectively, while those were 0.78 ± 0.92 and 0.49 ± 0.80 mm at 3 and 6 mm in the P(LA/CL) group. This tendency was similar to that in the collagen group. The absorbable ratios were 30.9 ± 23.9% and 31.8 ± 22.8% at 1 mm in the collagen and P(LA/CL) groups, respectively (Figure 4).

#### 3.4.2. Group B

The highest absorbance rate was found at 1 mm in the collagen and P(LA/CL) groups. The augmented bone widths were 2.67 ± 0.96 and 1.63 ± 1.28 mm at T1 and T2, and the absorbable ratio was 45.9 ± 43.5% at 1 mm in the collagen group. On the other hand, the augmented bone widths were 1.87 ± 0.56 and 1.44 ± 0.20 mm at T1 and T2, and the absorbable ratio was 19.9 ± 14.8% at 1 mm in the P(LA/CL) group. The absorbance ratio at all time points in the P(LA/CL) group was lower than that in the collagen group, although the difference was not statistically significant (the *p*-values of 1, 3, and 6 mm were 0.3228, 0.3063, and 0.8188) (Figure 4).

#### 3.4.3. Group C

**Figure 5:**
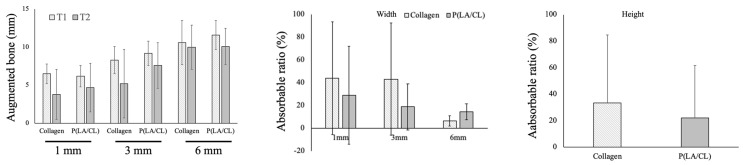
CBCT examination in group C.

The bone absorbable ratios were 44.2 ± 49.5 and 43.4 ± 49.2% (horizontally) at 1 and 3 mm, respectively, in the collagen group, while those were 29.1 ± 43.1 and 18.9 ± 20.1%, respectively, in the P(LA/CL) group. The horizontal bone absorption ratio in the P(LA/CL) group was lower than that in the collagen group, although this difference was not statistically significant. The bone height was decreased by 33.5 ± 51.2 and 22.0 ± 39.6% in the collagen and P(LA/CL) groups, respectively. There were no significant differences between the two groups (*p* = 0.7570) (Figure 5).

The augmented bone width was not significantly different between the two groups. The bone absorbable ratio of the P(LA/CL) group was lower than that of the collagen group but without significance. The height was maintained at almost the same level in both groups.

#### 3.4.4. All Groups

Change in augmented bone widths in all groups: in the collagen group, the absorbable ratios of the augmented bone were 40.9 ± 36.7, 29.4 ± 30.1, and 11.1 ± 22.0% at 1, 3, and 6 mm, respectively; the ratio at 6 mm was significantly lower than that at 1 mm (*p* = 0.0442). On the other hand, those were 26.2 ± 27.3, 17.1 ± 19.7, and 13.3 ± 16.4% at 1, 3, and 6 mm, respectively, in the P(LA/CL) group; there was no significant difference at any point (1 vs. 3 mm; *p* = 0.0952, 1 vs. 6 mm; *p* = 0.0747, 3 vs. 6 mm; *p* = 0.3826). No significant differences were observed between the 2 membranes at any time point (*p*-values of 1, 3, and 6 mm were 0.2617, 0.2254, and 0.7775, respectively) (Table 2).

### 3.5. Histological Analysis

**Figure 6:**
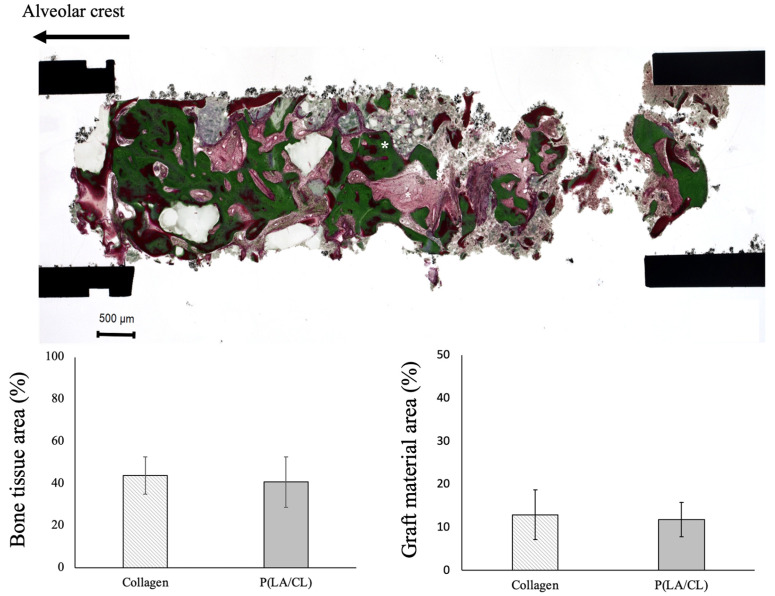
Villanueva-Goldner staining. The bone region was calculated by imaging software (* green; mature bone, dark red; osteoid).

The bone tissue (matured bone and osteoid) occupied 43.8 ± 8.8 and 40.8 ± 12.0% in the collagen and P(LA/CL) groups, respectively, in group C. In addition, the bone graft material occupied 12.9 ± 5.8 and 11.8 ± 4.0% in the collagen and P(LA/CL) groups, respectively (Figure 6). There were no statistically significant differences between the groups.

## 4. Discussion

The bone augmentation potential of GBR using the P(LA/CL) membrane, a biodegradable synthetic polymeric material, was comparable to that of GBR using a widely used collagen-based membrane. There was no difference in the resorption rate of the augmented bone width between the two membranes in group A. Although the resorption rates at 6 mm were almost the same between both membranes, those at 1 mm were 45.9 ± 43.5 and 19.9 ± 15% in the collagen and the P(LA/CL) groups, respectively, in group B (Figure 4). The absorption rates of collagen were >40% at 1 and 3 mm, whereas those of the P(LA/CL) group were approximately 20% at 1 and 3 mm in group C (Figure 5). The absorption ratio of the augmented bone tended to be lower in the P(LA/CL) group compared with the collagen group; however, the difference was not statistically significant. Although the sample size was small, the resorption rate of the bone augmented with the collagen membrane was higher than that of the bone augmented with the P(LA/CL) membrane. The particle size of the carbonate apatite used in this study was 0.6–1.0 mm, and it could make a large space between the particles. It was considered that the collagen membrane was absorbed before the maturation of the augmented bone; thus, it could not play a role in space-making. Subsequently, the immature augmented bone was absorbed under the pressure of the gingival mucosa. In contrast, P(LA/CL) played a space-making role until the augmented bone was almost completely mature. Consequently, the resorption rate of the augmented bone was at the same level as that of the bone augmentation with a titanium mesh [15]. Bone grafting materials with small particle sizes may induce better outcomes in the collagen membrane group.

The resorption rate of the augmented bone was lesser on the apex side compared with the apical side, regardless of the size of the defect. Furthermore, the larger the defect area, the greater the resorption rate. The tendency for less bone resorption in the P(LA/CL) group might be due to the characteristics of this material. P(LA/CL) is stretchable, and the barrier membrane is pinned with tension, consequently enhancing the stability of the graft material [16] in large defects, such as groups B and C in this study. The variation in data was significant in this study. This could be partially due to the small number of samples, ranging from 3 to 5 for each group, which could increase the number of individuals or regions. However, further studies are needed to confirm this hypothesis.

The ISQ values of the collagen and P(LA/CL) groups were 77.8 ± 3.3 and 79.2 ± 1.6 in group A, 74.9 ± 8.4 and 78.1 ± 4.0 in group B, and 80.0 ± 7.8 and 75.8 ± 4.3 in group C. All values were similar and were considered to be sufficient for osseointegration [17]. While the collagen-based membranes could carry a risk of unknown disease transmission because of the nature of the biological material, P(LA/CL) is a synthetic polymer with a low risk of unknown disease transmission. These results indicated that the P(LA/CL) membrane is safe and effective for GBR.

As a barrier membrane used in GBR, the biodegradable materials allow for a minimally invasive surgical procedure without the removal of the membrane. In this study, bone tissue occupied 43.8 ± 8.8% in the collagen group and 40.8 ± 12.0% in the P(LA/CL) group in the bone biopsy specimens. Although the bone biopsy specimen can include both the original and augmented bone regions, dental implants are usually placed in both the original and augmented bone regions to acquire osseointegration in clinical practice. The bone tissue occupancy was reported to be 20–73% in the alveolar bone augmentation procedure after tooth extraction [18], and approximately 40% in GBR [19]. The bone graft material occupied 12.9 ± 5.8 and 11.8 ± 4.0% in the collagen and P(LA/CL) groups, respectively; in this study, the ratio of the residual graft material was almost the same between the two groups. Nakagawa et al. [20] reported that the new bone and carbonate apatite areas were 33.8 ± 15.1 and 15.3 ± 11.9%, respectively, in the bone biopsy which was performed 8 ± 2 months after the maxillary sinus floor augmentation with carbonate apatite. These data were consistent with our results. Thus, reasonable bone regeneration was achieved using carbonate apatite and collagen- or P(LA/CL)-based membranes for GBR.

Collagen-based materials are the most commonly used biodegradable barrier membranes in GBR. Although collagen has excellent biocompatibility [21], it lacks graft material stability due to its low-grade strength under pressure from the mucosa when used as a membrane in GBR [5]. Furthermore, collagen has the disadvantage that the GBR would induce an insufficient outcome when the membrane is exposed to wound rupture [22], partially due to bacterial infection. This is because the collagen membrane has large enough pores for bacterial infiltration. In contrast, P(LA/CL) membranes have a unique dual-layer structure [13]. The outer layer was solid and inhibited bacterial infiltration [23]. Therefore, wound rupture was not considered to have led to infection caused by bacterial contamination. 

One disadvantage of many synthetic polymers is their unpredictable biodegradability. An inadequately short period of membrane biodegradation would induce insufficient bone augmentation, whereas prolonged residual material would necessitate its removal. P(LA/CL) has been reported to be absorbed and degraded in 12–26 weeks [13]. No material remained clinically or histologically for at least 150 days postoperatively (approximately 20 weeks). Considering the period of bone augmentation by GBR, it is desirable that P(LA/CL) does not remain long enough to elicit undesirable clinical outcomes.

Another disadvantage is the unpredictable resorption of the augmented bone with GBR after surgery [24]. This study also showed that the resorption rate of the augmented bone volume varied widely in both the collagen and P(LA/CL) groups (Figure 4 and Figure 5), indicating the difficulty in predicting the amount of augmented bone absorbed after GBR. This issue should be addressed in future studies. 

A limitation of this study was the small sample size; consequently, a future study with a larger sample size is necessary. Moreover, the change of augmented bone volume needs to be evaluated over the long term because the long-term change of the augmented bone by GBR remains controversial [25,26].

## 5. Conclusions

The P(LA/CL) membrane demonstrated a bone augmentation potential similar to that of the collagen membrane in GBR. P(LA/CL) is a synthetic polymer that can, therefore, be used as a safe and effective membrane in GBR.

## Figures and Tables

**Table 1:** The list of groups.

Group	Total Sites (Number of Patients)	Collagen (Number of Patients)	P(LA/CL) (Number of Patients)
A	7 (7)	3 (3)	4 (4)
B	10 (7)	4 (4)	6 (3)
C	8 (6)	3 (3)	5 (3)
Total	25 (20)	10 (10)	15 (10)

**Table 2:** Absorbable ratios (%) of the augmented bone width in all groups.

	1 mm			3 mm			6 mm		
	T1 (mm)	T2 (mm)	Absorbable Ratio (%)	T1 (mm)	T2 (mm)	Absorbable Ratio (%)	T1 (mm)	T2 (mm)	Absorbable Ratio (%)
Collagen	3.8 ± 2.1	2.3 ± 2.0	40.9 ± 36.7	4.3 ± 3.1	2.9 ± 2.7	29.4 ± 30.1	4.7 ± 4.4	4.4 ± 4.2	11.1 ± 22.0 *
P(LA/CL)	3.4 ± 2.2	2.6 ± 2.4	26.2 ± 27.4	5.0 ± 3.2	4.1 ± 3.1	17.1 ± 19.7	6.1 ± 4.2	5.3 ± 3.8	13.3 ± 16.4

* The absorbable ratio was significantly different between 1 and 6 mm in the collagen group (*p* = 0.0442).

## Data Availability

Not applicable.
